# Interpersonal determinants of eating behaviours in Dutch older adults living independently: a qualitative study

**DOI:** 10.1186/s40795-020-00383-2

**Published:** 2020-11-11

**Authors:** Andrea Johanna Bukman, Amber Ronteltap, Mila Lebrun

**Affiliations:** 1 Knowledge Centre Healthy and Sustainable Living, University of Applied Sciences Utrecht, P.O. box 12011, 3501 AA Utrecht, The Netherlands; 2 Alimentation Science Department, Danone Research, Palaiseau, France

**Keywords:** Eating behaviour, Older adults, Interpersonal determinants, Qualitative research

## Abstract

**Background:**

Eating behaviour of older adults is influenced by a complex interaction of determinants. Understanding the determinants of a specific target group is important when developing targeted health-promoting strategies. The aim of this study was to explore interpersonal determinants of eating behaviours in older adults living independently in a specific neighbourhood in the Netherlands.

**Methods:**

In the neighbourhood of interest, populated by relatively many older adults, fifteen semi-structured interviews were conducted with independently living older adults (aged 76.9 ± 6.4y). Interviews were complemented with observations among the target group: three occasions of grocery shopping and three collective eating occasions in the neighbourhood. A thematic approach was used to analyse the qualitative data.

**Results:**

When we asked the older adults unprompted why they eat what they eat, the influence of interpersonal determinants did not appear directly; respondents rather mentioned individual (e.g. habits) and environmental factors (e.g. food accessibility). Key findings regarding interpersonal factors were: 1) Behaviours are shaped by someone’s context; 2) Living alone influences (determinants of) eating behaviour via multiple ways; 3) There is a salient norm that people do not interfere with others’ eating behaviour; 4) Older adults make limited use of social support (both formal and informal) for grocery shopping and cooking, except for organised eating activities in the neighbourhood. In this particular neighbourhood, many facilities (e.g. shops at walking distance) are present, and events (e.g. dinners) are organised with and for the target group, which likely impact (determinants of) their behaviours.

**Conclusions:**

The study showed that older adults do not directly think of interpersonal factors influencing their eating behaviour, but rather of individual or environmental factors. However, multiple interpersonal factors did appear in the interviews and observations. Moreover, neighbourhood-specific factors seem to play a role, which underlines the need to understand the specific (social) setting when developing and implementing intervention programmes. Insights from this study can assist in developing health-promoting strategies for older adults, taking into account the context of the specific neighbourhood.

## Background

The proportional rise in the ageing population, particularly in Europe [1], increases the need for healthy ageing. A healthy diet is recognized to be important for successful ageing. Healthy diets among older adults are associated with better health and quality of life [2], and lower risk of mortality [3]. However, for older adults it can be difficult to eat healthily, for example, because of physical problems or a reduced appetite [4, 5], and therefore they can become at risk of malnutrition [6]. Malnutrition is a threat to the health, autonomy, and well-being of older adults [7]. A frequently observed problem is a decline in energy and protein intake [7]. Interventions promoting a healthy diet can improve eating behaviour of older adults [8, 9]. For promoting a healthy diet among older adults, it is important to not only focus on nutrient intakes (e.g. protein, vitamin D), but also to take the context of eating into account, making nutritional recommendations more relevant to people’s everyday life [10]. The ageing process is involved with several changes (e.g. widowhood, onset of disease) that influence people’s context of eating, and that should be taken into account when trying to promote healthy diets [7]. Therefore, it is important to understand the determinants of this target group’s eating behaviour.

Generally, determinants of nutrition and eating can be subdivided into factors at an individual, interpersonal (social and cultural), environment, and policy level [11]. A review study has shown that food choice of older adults is influenced by a complex interaction of determinants; the study describes many determinants, most of which at an individual level (e.g. poor dentition, loss of appetite, mobility/functional limitations, lack of motivation and/or energy, skills in food preparation, and income) [12]. Several other studies described the importance of interpersonal determinants in older adults’ eating behaviour, such as social influence (e.g. presence of others, social norms) [12–14], and social support (e.g. formal support, social ties) [12–17]. Also, the absence of others, for example, when living alone or being widowed [12, 13, 15–17] has been shown to influence eating behaviour of older adults.

Although there is considerable knowledge about different factors affecting eating behaviours of older adults, general insights into factors affecting dietary behaviour of older adults will likely be inadequate to optimally align intervention programmes to local settings. It is vital to understand the exact meaning of these determinants in the everyday life of targeted older adults in their specific contexts, as we also know that determinants of eating can differ between contexts [18–21]. Deeper understanding of the perceptions of the target group will help to develop suitable strategies to promote healthy eating. The aim of the current study was to grasp the interpersonal determinants of eating behaviour in older adults living independently in a specific area in the Netherlands (Lage Land area, Rotterdam).

## Methods

To gain a deeper understanding of interpersonal determinants of eating, older adults were interviewed, and grocery shopping and collective eating occasions were observed. Complementing interview studies with observations was recommended in previous research as it can help to detect issues that may not directly come up in interviews [12]. The current study served as a pre-study; the complete study, including this pre-study, was evaluated by the Medical Research Ethics Committee of University Medical Center Utrecht. The committee decided that the Dutch law concerning Medical Research Involving Human Subjects Acts does not apply to the study, and, therefore, official approval is not required. To contribute to explicit and comprehensive reporting of qualitative studies, we completed the COREQ 32-item checklist (see Additional file 1).

### Context of study

The current study served as a pre-study for a multicomponent group-based fall prevention programme targeting older adults [22]; participation in the pre-study was unrelated to participation in the fall prevention programme. Insights from the pre-study served as input for targeting the nutritional component of the programme towards older adults living in the specific neighbourhood of interest. The neighbourhood of interest was a neighbourhood in the city of Rotterdam, which is one of the largest cities in the Netherlands. In the neighbourhood of interest, 23% of the inhabitants were 65 years or older compared to 15% in the city as a whole [23]. Socioeconomic status was comparable to that of the rest of the city [23].

### Interviews

Fifteen semi-structured individual interviews were conducted with fifteen participants. Fourteen of them were personally recruited (face-to-face or by telephone) by either a local welfare worker or a dietician, both involved in the fall prevention programme; one participant was recruited at a collective eating activity in the neighbourhood (also see next heading: Participant observations). More than half of the older adults who were invited were willing to participate in an interview; reasons for non-participation were a poor health status or no interest. The interviewer and interviewee did not know each other before the start of the study. Inclusion criteria for the interviews were: aged 65 years or older, living independently, and living in the specific geographic area of interest. In addition, as this was a pre-study for a fall prevention programme, the professionals who recruited participants were asked to select individuals with a presumptive increased risk of falling (e.g. indicated by experienced falls in the previous year, balance impairment, having difficulties walking). However, this criterion appeared to be complicated to apply, for example, because fall-proneness was not registered in their database or because professionals found it awkward to ask. As fall risk was not the main focus of the present study, we did not screen participants ourselves; hence, we cannot be certain whether they had an increased risk of falling. However, it is likely that our sample had impaired vitality (e.g. indicated by using a wheeled walker).

The average age of the participants was 76.9 ± 6.4 years (range = 68–89 years). Four males and eleven females participated. Marital status varied: five participants were widowed, two participants were married, five participants were divorced, and three participants were unmarried, of whom one was in a long-lasting relationship. The three participants with a relationship lived together with their partner. The participants without a partner lived alone, except for one woman who lived with her child. During the interviews, many participants mentioned their former profession; based on those professions, it can be concluded that also socioeconomic background varied among our sample.

The interviews started with some general open questions to become familiar with each other and create an atmosphere of trust, including questions on: socio-demographics, daily activities, and spontaneous associations with food and eating. Furthermore, the interview guide (see Additional file 2) included the topics: food choice motives, traditions, habits, definition of a proper meal, skipping meals, eating alone and/or with others, grocery shopping, meal preparation, formal meal preparation support, social ties, loneliness, and changes in diet. The selection of topics in the interview guide was based on literature related to interpersonal determinants of eating behaviour of older adults [11–17, 24]. The interviews were prepared following the interview guideline of Baarda [25]. After the first interview, one question was added to the interview guide about telephone contact as part of social ties.

The interviews took about 1 hr and were held at a location of the participant’s choice which was either at their homes (*n* = 3), or a separate room at the local community centre (*n* = 12). The interviews were conducted by one of four researchers of the University of Applied Sciences Utrecht (including the authors AJB and AR, both female, and two male colleagues), assisted by a second researcher from the project team. The second researcher observed and made notes. Participants gave written informed consent before the start of the interview. All interviews were audio-recorded and transcribed verbatim. Interviewees were not asked for additional information afterwards.

Data were analysed thematically. The authors AJB and AR created the initial code scheme after reading transcripts of the interviews, and tested the code scheme by coding all interviews. After discussing the coding process, one code was added, and other codes were clarified in more detail; resulting in a code tree consisting of nineteen main themes and 35 subthemes. The final coding of all interviews was done by one researcher (AJB). The data were manually coded in the program Atlas.ti 8. The analysis started by discussing initial insights among all researchers involved. Then, AJB started analysing the coded data, starting with the codes that came up from the discussion, which were ‘paying attention to or talking to others about eating behaviour’, and ‘loneliness’ in combination with ‘being alone’. Next, text fragments belonging to related codes were analysed in order to link them to each other. Finally, all coded text fragments were analysed, to prevent missing relevant insights. Insights were discussed with AR and ML, and, eventually, four key topics were identified in relation to interpersonal determinants of eating. The findings were compared with the results of the observations, checked with the other interviewers, and supported with illustrative quotations (translated from Dutch to English). To ensure the anonymity of respondents, the interview where the quotation was extracted from is not reported. When respondent characteristics were relevant for interpretation (e.g. marital status), the characteristic is stated between brackets following the quotation. All fifteen interviewees are quoted at least once in the results section and themes were always a result of data from multiple interviews.

### Participant observations

Observations were done at three shopping locations (five observations) and three collective eating activities (five observations) in the neighbourhood. The shopping locations were two supermarkets and the weekly local market. The eating activities included one lunch occasion and two dinner occasions. The eating activities were organised by local welfare organisations with the help of volunteers (mostly older adults), and often visited by older community members. The activities took place at central locations in the neighbourhood (e.g. community centre). The number of participants per occasion varied from 20 to 30 persons. Some participants did know each other, others did not. Some visited the eating occasions weekly, others less often. Participants paid €2.50 for their lunch and €5.50 for their 3-course meal. Participants at the eating activities were informed about the purpose of the participant observation, and subsequently gave us permission to join. Researchers used an observation form to make notes that could not be traced back to individual participants. The same four researchers (including the authors AJB and AR) as during the interviews led the observations. The observation form included the topics: type of behaviour, conversations, description of context, description of target group, mood, other areas of observations and reflective comments; as adapted from Roller [26]. Insights from the participant observations were coded based on the themes that came up from the interviews. The combined insights, based on theme rather than data source, are presented (see Table 1).

**Table 1:** Themes and data sources

Theme	Related codes from interviews	Related data from observations
1) Behaviours are shaped by someone’s context	Habits / traditions Food motives: - Habit / routine / used to (from the past) - Going along with the social context / someone else chooses	Not directly observed. However, during the collective eating activities, there was limited choice in what to eat, and participants ate whatever was served (going along with the context).
2) Living alone influences (determinants of) eating behaviour via multiple ways	Food motives: - Price / budget - Easy / quick / saving energy (dishes) - Living alone / feeling alone Social context of a meal Loneliness	From conversations during collective eating activities: - The influence of living alone on life. - Sociability as a reason to attend the collective eating activity.
3) There is a salient norm that people do not interfere with others’ eating behaviour	First association / interest eating and drinking Food motives: - Because someone else tells me (not) to Paying attention to the behaviour of others / talking with other about eating and drinking (Not) comparing own behaviour with others	From conversations during collective eating activities: - Some older adults did seem to pay attention to others’ eating behaviour during the activity. - The few conversations about eating were superficial, mostly about the foods on the table. - Seeing or hearing about certain foods could raise someone’s interest to try it themselves (e.g. to try a meal service that someone else was talking about).
4) Older adults make limited use of social support (both formal and informal) for grocery shopping and cooking, except for organised eating activities in the neighbourhood	Informal support with meal preparation Formal support with meal preparation Grocery shopping	Observed while visiting the collective eating activities and the occasions of grocery shopping: - Multiple facilities nearby - Many older adults make use of it From conversations at the weekly market: - The weekly market was not only visited for grocery shopping, also to have a walk outside.

## Results

When we asked the older adults unprompted why they eat what they eat, the influence of interpersonal determinants did not appear directly; respondents rather mentioned individual and environmental factors. Their first response was generally that they eat what they are used to eat.

*“Well, I have this fixed thing, every morning a sandwich. With apple syrup. Along with a cup of tea. And then I have two cups of coffee. Around 12, or just after 12, I take a sandwich, or two crackers, with cheese.”*

They explained that their eating pattern just works for them; it is something they discovered in the past, either because of their health, because they feel hungry at those times, or because of the ease of preparing it. Respondents also mentioned the influence of other individual factors on their food choices, like taste preferences, (changes in) health status, medication use, and weight loss intentions. An often-mentioned environmental factor was the availability of products, both at home and in shops.

Interpersonal factors were not top of mind, but did play a role in respondents’ eating behaviour in different ways. Results from multiple code groups combined led to the emergence of four main themes with respect to interpersonal factors: 1) Behaviours are shaped by someone’s context; 2) Living alone influences (determinants of) eating behaviour via multiple ways; 3) There is a salient norm that people do not interfere with others’ eating behaviour; and 4) Limited use of social support (both formal and informal) for grocery shopping and cooking.

## Behaviours are shaped by someone’s context

Several respondents said that social context – both current and previous – influenced their eating behaviour. More specifically, social context influenced *what* they eat and *how much* they eat.

Some respondents indicated that they go along with their social context, in terms of what they eat. For example, they eat whatever someone else cooked:

*“You just eat what’s cooked, haha!”*

Or they adapt to what a partner prefers to eat:

*“Well I always ask [my partner] what are we eating tomorrow, or what do you want to eat? And then I’ll go get it the next day.”*

Having visitors is mentioned as a reason to cook something special and/or more elaborately.

*“Because when my grandsons, when they come, then one says: gran, I want to have fried rice. Then I will make fried rice, extensively, for them.”*

*“It is, someone comes over for dinner, then I do it more extensively of course than when I am in that kitchen by myself.”*

Eating together is mentioned by one respondent as a reason to eat more:

*“And you’re in a large group and everybody eats, and then you eat more than you planned. Then you can’t stick to one sandwich with cheese.”*

The influence of others on older adults’ eating behaviour can also be derived from their cultural background, i.e. religious or family habits. They, for example, retain traditional habits for a certain day of the week:

*“Maybe that has to do with the past or something. I don’t know. Yes, that uh … Some things I just don’t eat on Sundays on principle. Very strange, but … [ … ]. Well, in the past, there wasn’t … wasn’t much money so you didn’t really eat meat, that happened sometimes. Yes, and apparently that is ingrained or something, I don’t know.”*

*“Yes the two days before Easter. Yes yes. No meat, fish on Good Friday. That stems from religion right, the Catholic religion.”*

Or for a specific situation:

*“Yes, soup day. Or not directly soup day, but yes sometimes soup. When people are ill, a cup of soup.”*

Or more generally, their attitude is influenced by the way they were raised:

*“Noooo. We learned of course … like in the old days, you eat what’s on the table. [ … ] That is still old-fashioned. I never complain about the food.”*

A change in social context, such as divorce or widowhood, also influences eating behaviour. For example, a change in family composition can influence the effort someone puts in meal preparation (also see next theme: living alone), and can also influence the enjoyment of eating:

*“I didn’t like it, well, I do when I eat with other people of course. No, not alone. That isn’t … I can’t get used to that. I couldn’t get used to that. [ … ] Yes, and you didn’t have inspiration to cook something, you didn’t feel like it. Look, you gotta eat, so you had to make something, but it just lost its savour.”*

## Living alone influences (determinants of) eating behaviour via multiple ways

Living alone, whether or not after losing a partner, was often mentioned by respondents as a reason to explain their choices. The absence of others influences the extent to which shopping, preparing, and serving food is perceived to be worthwhile. From our data, multiple ways emerged through which being alone may explain eating behaviour.

### Effort worthwhile

Several respondents who live alone indicated to make food preparation easy for themselves, for example, by cooking multiple portions of a meal at once, cooking one-pot meals, or buying ready-to-eat meals.

*“I did cook, uh yes even extensively sometimes, and then I had a huge pile of dishes, and I thought well I’m not doing that again. Dear no.” (divorced respondent)*

*“I make things as easy as possible for myself. Because then I don’t have to cook. So I save that energy. I have less dishes, also energy I save.” (unmarried respondent)*

As described before, living alone after a divorce or losing a partner, can influence the effort someone puts into a meal. Respondents who used to live together with someone else, but now live alone, described how they put less effort into a meal than before:

*“Yes, and if you’re alone then uh, you don’t really uh make a fuss anymore.” (divorced respondent)*

### Price

When asked whether price influences their food choices, several respondents brought up the influence of being alone on their perception of price. For example, some respondents said that when being alone, price could be less of an issue since cooking for one person is not perceived as expensive.

*“Well, look, yes, I don’t really consider that because when I’m alone it all don’t have to be that expensive right?” (divorced respondent)*

On the other hand, cooking for one person can be perceived as expensive when making a more elaborate meal, as this involves multiple ingredients:

*“Well dinner for one person … that’s not too expensive. [ … ] It is of course if you’re making something elaborate and you are alone, then it’s troublesome, for one person. Or else you cook for 2 days, but still. You need so many ingredients you know. Then it is expensive all right.” (divorced respondent)*

### Eating out to seek company

Eating out with others, instead of eating alone, was described as more enjoyable. As noticed during the participant observations and the interviews, multiple respondents make use of collective eating activities in the neighbourhood because of reasons of sociability.

*“Yes I like that. Because then we’re with a group at the table of course. That’s more sociable than eating alone.” (widowed respondent)*

The social aspect seemed to be even more important than the food itself:

*“But I don’t do that to eat out, but to have contact with people.” (unmarried respondent)*

*“Well, that you, those contacts … actually, because it’s always a surprise what you eat, so it’s not that I think at that moment I’m going out for a nice dinner tonight, I’m going there. No, it’s just we’ll just see there and I’ll see whatever they’ll be eating.” (divorced respondent)*

From the interviews, the impression arose that especially people who live alone make use of the collective eating activities. During the observations, there were also some couples who participated in the organised diners. Also for them, sociability was a reason to go to collective eating activities.

## The salient norm that people do not interfere with others’ eating behaviour

The data revealed a salient norm to only pay attention to your own eating behaviour, and not to discuss it with others, let alone comment on other people’s eating behaviour. Respondents indicated that eating is not a topic of conversation, although sometimes it is discussed in relation to health, or with health professionals.

### Paying attention to each other’s eating behaviour

Initially, most respondents indicated that others do not pay attention to what they eat. However, over the course of the interview, it became apparent that dieticians or other health professionals had provided them with dietary advice at some point. Some respondents had a partner or friend who occasionally commented on their dietary behaviour:

*“ … something my wife keeps going on about a bit, is this fruit.”*

*“And at a certain point my friend said, this was May last year, she says ‘gee, you are getting a round chubby head’.”*

Vice versa, respondents indicated not to pay attention to what others eat:

*“Couldn’t care less what they eat.”*

*“Everyone needs to decide for himself whatever they take or what they eat.”*

Although most interview respondents said not to pay attention to other people’s eating behaviour, multiple remarks show that people *do* notice others’ behaviour. In addition, the observations of collective eating occasions revealed that some participants did seem to pay attention to others’ eating behaviour. For example, one participant commented on another’s addition of salt. What seems to lie underneath is a felt norm that people should decide for themselves what they eat:

Respondent*: “Then I see the difference that they pile up (the sandwich filling), right. I find that strange. But then I think, well, so that’s their way of eating.”* Interviewer*: “So you notice it, but you don’t do anything with it?”* Respondent*: “No, why would I? Usually I don’t pay attention.”*

*“Yes, well maybe I do pay attention to it, but it’s up to them. I’m not going to be uh … miss know-it-all, right? Haha! [ … ] let’s say if someone eats something that’s not quite right, I’m not gonna say should you be doing that or something. Uh yes, you know what’s right or wrong right?”*

When asked how healthy respondents eat compared to other older adults, many respondents answered that they usually do not compare themselves or do not know what others eat. If they do compare themselves, there is variation with whom, and on which type of behaviour (e.g. portion size, skipping meals, and medication use).

*“I don’t relate to that. I don’t do that ( … ) I am above that. Everyone just needs to do things however they want.”*

*“Yes you know, I don’t pay much attention to the people around me, what they eat. I don’t ask either.”*

*“I don’t know, I never really talk about that. Everyone should figure that out for themselves. No, I’m not gonna check out someone else’s plate to see what they … they should sort that out among themselves.”*

Some respondents even do not associate themselves with older adults:

*“But for the rest, I don’t pay attention to it, I … I don’t really have much contact with older people. I don’t really like older adults. Haha.”*

*“There aren’t any [of my age]. Certainly not here. They are older than me or much younger than me.”*

### Talking about eating

Respondents not only claimed not to pay attention to others’ eating behaviour, they also indicated to hardly talk about eating. The rare occasions they do talk about eating are rather superficial, e.g. about foods they like, or what they plan to eat for dinner.

*“No. It’s always: it’s tasty again. Yes. Well. That’s it. It’s tasty again, that’s the only thing you talk about. For the rest, you talk about other things.”*

*“Well, well yes with my friend, if we come back from course in the morning, we sometimes talk about it like: gee, what are you eating tonight? And then she says this or that and then I say that … but that’s not the main issue. No. Actually very brief.”*

These results were confirmed during the observations of eating occasions; the few conversations about eating were superficial, mostly about the foods on the table (e.g. whether respondents liked the food, or whether they had had it before). The conversations were rarely about their eating behaviour more broadly.

The fact that older adults do talk to each other about eating sometimes, can also be concluded from the finding that some respondents indicated becoming inspired by others to try something new, for example meal services, or specific new food products.

*“Yes I already knew that. I had a neighbour beside me. A gem. And this butcher visited her every week already.”*

*“Look I had quinoa at home. I got it from my neighbour here. So I said I’ll try it out. [ … ] If I didn’t have it I wouldn’t have done it! But I got it, so I thought I’m not gonna throw it away. I’ll just try it.”*

Also during the observations, some older adults were inspired by others. Someone was inspired to try a meal service that someone else was talking about, and two others were discussing that the soup that was served was something they could try to make at home.

## Limited use of social support (both formal and informal) for grocery shopping and cooking

The willingness to help each other in this particular neighbourhood was mentioned more than once, both during interviews and observations. Generally, respondents did not make much use of support in grocery shopping and cooking, except for the organised eating activities in the neighbourhood, and, occasionally, formal support in the form of ready-to-eat meals.

### Grocery shopping

Most respondents did their grocery shopping themselves. Sometimes their partner bought the groceries. Only one respondent mentioned that they purchased most of their groceries online:

*“Well, I’m not gonna lug anymore. We used to have a car. We went for the groceries ourselves. But I’m not doing it anymore, can’t carry it anymore. And my husband aged too … he goes to the market and is able to get one or two things, but he doesn’t have to lug. The boys from [supermarket] bring everything.”*

The interviewed older adults mostly did their groceries without any help, although they sometimes had neighbours offering help.

*“No, I do that myself. All by myself. Until now always by myself. The neighbours like to come around. One always asks: I’m going to the [supermarket] or I go to [the other supermarket] now, do you need anything? I say: well no.”*

Not all respondents seemed happy with the lack of support:

*“Well I was disappointed at my birthday, that none of my children asked me: do you need anything?”*

*“No that’s ok. Well there are things sometimes, for example potatoes are 3 or 4 kilos, that’s less nice. That’s annoying. But well, that’s the way it is. It’s no different. The fairies won’t do it, I always say.”*

However, most interviewees seemed to prefer to do their own grocery shopping, for which they gave different explanations. One reason is that older adults want to stay independent.

*“No then you’ll probably have your groceries delivered to your home. But I wasn’t planning on doing that for a little while. I want to stay independent as long as possible.”*

Another possible reason could be that they do want to select their own products.

*“Last year uh, a very sweet couple down the hall here always got it for me. But then it’s not quite what you’d like to have. And now I do everything myself again.”*

Lastly, doing their own grocery shopping can also be a way for older adults to get out of their homes, or to take a walk.

*“I try to go outside for a little while every day. If only to get one little thing, then at least you’re outside for a little walk.”*

*“I walk there. [Supermarket] is my favourite shop. They are all very nice there. And some of them know what I have. Then every now and then they ask: and how are you? You know things like that.”*

The observations confirmed this result; some of the older adults at the weekly market indicated they were not there to buy groceries, but to have a walk outside.

The presence of many shops and a weekly fresh market in this neighbourhood facilitates the older adults in buying their own groceries. Moreover, there are good public transport options.

*“Stumble twice and I’m in.”*

### Meal preparation

Mostly, meals were either cooked by older adults themselves, or by the people they live with. On rare occasions, a neighbour or relative came over to help. Some respondents did make use of organised eating activities in the neighbourhood, or ready-to-eat meals.

For some respondents, formal support in meal preparation was provided by the local butcher, who delivers freshly made, ready-to-eat meals at home. Most respondents were familiar with this particular butcher’s. These ready-to-eat meals were considered good meals by many respondents, because of the taste and freshness, in contrast to regular ready-to-eat meals available in supermarkets.

*“At the butcher’s. Not at [supermarket] these meals, then I’m looking and then my sister sometimes says, this or that nice ready-to-eat meal, well that’s good food, she says. And then I say, well yes, I’m a bit of a fussy eater anyhow. Yeah I don’t know. Is it fresh? It has been packaged for a long time, sometimes it’s all moist, you know, from condensation or something. Imagine me eating that, no I won’t eat it.”*

However, many respondents reject ready-to-eat meals whatsoever, as long as they can cook themselves.

*“But I don’t think that’s necessary yet. As long as I can I will do it myself.”*

*“I can, as long as I can. You know. Perhaps when I get older and can’t take care of myself anymore. And I become dependent, yes then maybe I should do it too.”*

Ready-to-eat meals were associated by respondents with being not tasty, too salty, and less fresh. Most of them knew people who eat ready-to-eat meals, and, as such, they sometimes tried it too as it can be convenient sometimes.

*“Yes my age group, I know, lots of single people buy these meals. Terrible but they’re not tasty.”*

An organised eating occasion could be an alternative to ready-to-eat meals. There are many social (eating) events for older adults organised in the neighbourhood.

*“I’d rather, when the time has come, go to, what are these things called, you have these..., yes not really older adults’ homes there, but they all have these kinds of restaurants nowadays, don’t they?”*

As described earlier, sociability is a frequently mentioned reason by older adults to go there. However, an additional advantage, as mentioned by one respondent, is that you do not have to cook yourself.

*“You know, then I don’t have to cook myself. That’s just the way it is.”*

## Discussion

This study aimed to gather insight into interpersonal determinants of eating behaviour of independently living older adults, in a specific neighbourhood in the Netherlands. The study showed that older adults do not directly think of interpersonal factors influencing their eating behaviour, they rather think of individual factors (like habits and health status), or environmental factors (like food accessibility). However, interpersonal factors did seem to influence their eating behaviour as well. With respect to interpersonal factors, we identified four key topics: 1) Behaviours are shaped by someone’s context; 2) Living alone influences (determinants of) eating behaviour via multiple ways; 3) There is a salient norm that people do not interfere with others’ eating behaviour; 4) Limited use of social support (both formal and informal) for grocery shopping and cooking, except for organised eating activities in the neighbourhood.

Some results seem neighbourhood-specific. It was striking how many social events for older adults were organised in the neighbourhood, probably due to the relatively large number of older adults in the neighbourhood; 23% of the neighbourhood’s inhabitants were 65 years or older, compared to 15% in the city as a whole [23]. In the neighbourhood, there are many shops, a weekly fresh market, and good public transport options. Those facilities can explain why the older adults in this neighbourhood did not make much use of social support for their grocery shopping. The norm not to interfere with each other’s eating behaviour is probably less neighbourhood-specific, but rather part of Dutch norms in general. Literature shows that for some, like the French, eating is a social matter, where for others, like the Americans, eating is rather an individual matter, or even personal freedom or responsibility [19]. For the Dutch, it is likely that eating is perceived more as an individual affair, as the Netherlands is more individually than collectively oriented [27, 28], and individual responsibility regarding health and lifestyle are considered important [29].

Other findings of our study were expected to be more generally applicable to older adults; for example the influence of living alone [12, 30, 31]. Although living alone is sometimes classified as an individual determinant of eating behaviour [11], respondents in our study described the influence of living alone on their eating behaviour in terms of the absence or presence of others. Therefore, we included living alone as one of the relevant interpersonal factors. Eating alone is associated with a reduced diet quality [32, 33]. In our study, living alone was often mentioned by the respondents as a reason to explain their food choices. The finding that they put less effort into food preparation when they are alone, is in line with other studies that showed that living alone is associated with simplified meals and less food diversity [30, 34]. Likewise, some community-dwelling older adults in the study of Van der Pols-Vijlbrief et al. [31] mentioned that eating alone causes a lack of motivation to cook nutritious meals. Moreover, some of our respondents explained that eating alone is less enjoyable, which is also in line with other studies that showed that eating alone reduces the pleasure of eating [12], and food is perceived tastier when eating together [31].

Also more generally applicable is the finding that older adults’ eating behaviour is partly led by their context. Some of our participants’ eating habits were a result of religious or (earlier) family traditions. Earlier studies described that present life of older adults is affected by habits founded in the past [35, 36]; however, these traditions can also slightly change when getting older, for example, because people become dependent on others for their groceries [34, 37]. Also, norms and preferences of others in the current social context can influence older adults’ eating behaviour, as some respondents indicated going along with their context.

One strength of this study is that it included both interviews and observations. The observations mostly confirmed the findings from the interviews and helped to better understand the perceptions and experiences that older adults described during the interviews (for example, where they did their groceries, and the eating activities they attended). It also helped to detect incidental discrepancies between what was expressed by the older adults, and what was observed by the researchers. An example of such a discrepancy is that interview participants indicated not paying attention to each other’s eating behaviour, while the observations provided some concrete examples of occasions where they did.

We aimed to interview a diverse group of older adults, to gain insight into the different perceptions that may exist among older adults in the neighbourhood of interest. Our group was diverse in terms of – among other things – age and marital status, but not in gender; the majority was female. The small number of males included in this study, may have caused some male-specific perceptions not to be detected. Moreover, there might have been selection bias as a result of recruiting participants via local welfare and health professionals. The perceptions of older adults who are not in sight of these professionals may have been missed. Moreover, the older adults involved in our study were possibly less vital than the general population of older adults, as professionals attempted to recruit interview participants with an increased fall risk (e.g. indicated by experienced falls in the previous year, balance impairment, having difficulties walking). It is known that impaired mobility can be a barrier for shopping and meal preparation [12], for which older adults then have to rely on others [38]. Therefore, selecting on increased fall risk may have influenced the findings of the current study (e.g. because interview respondents were more in need of social support than other older adults). The increased fall risk did not, however, inhibit most older adults included in our study from doing their own grocery shopping, or coming to the community centre for the interview. The observations at the supermarkets and local market helped us to get into contact with a more diverse group of older adults. In conclusion, the current methods cannot guarantee that we gained an exhaustive view of interpersonal determinants in the area of interest. This study did provide valuable insights in both neighbourhood-specific and general determinants of older adults’ eating behaviour, and particularly in the meaning of the determinants for the older adults in their daily lives.

The current study focused on interpersonal determinants. As shown in literature, determinants at an individual, environmental, or policy level will also influence eating behaviour [11]. Hence, for health promotion, it is important to consider factors at other levels as well. In our interviews, those other factors appeared too, e.g. habitual eating, taste preferences, or food availability. It would be interesting to further explore the interaction between interpersonal determinants and these factors at other levels, influencing eating behaviours. In our study, for example, some respondents described that the presence of many shops (an environmental determinant) in this neighbourhood facilitated buying their own groceries, which made them less needy for social support in grocery shopping. Another example of such interaction, from literature, is that receiving social support for grocery shopping or meal preparation can conflict with one’s own taste preferences (an individual determinant) [14, 39]: the people who provide support may use different products or cooking techniques. This was not the case in our study: participants did not make much use of social support and were therefore probably able to follow their own food preferences. So as long as their health status (an individual determinant) enables them to continue their habitual behaviour (another individual determinant), social support for grocery shopping will not influence their eating behaviour much. Likewise, as the norm was not to interfere with others’ eating behaviour, these others will not be a reason to change behaviours, which could further reinforce their habitual behaviours (an individual determinant). Detailed knowledge of these possible interactions can further inform health promotion programmes.

The insights from the current study assist in developing health-promoting strategies for older adults. In general, it is necessary to recognize the strong influence of habits (partly founded in the past) by providing advice that is in line with older adults’ own dietary patterns, and the underlying norms and preferences. The influence of living alone on the effort they are willing to spend on cooking elaborately should be considered, by providing solutions that do not require much effort in meal preparation. Social eating can be a solution for some older adults; when eating is combined with another activity the target group may be more likely to go [40]. When eating is not so much a topic of conversation for them, as in the neighbourhood of our interest, this other activity should be focused on something else, or be promoted by emphasising the social aspect of being with others (but not interfering with each other’s behaviour). The benefits of an adequate diet for staying healthy and independent can be emphasised, as these can be strong motives for older adults. Careful consideration is necessary regarding the framing of ‘older adults’ in (the recruitment for) the programme, as, in line with our finding, they do not necessarily identify themselves with this group [40].

## Conclusions

The current study provided a deeper understanding of interpersonal factors in eating behaviour according to older adults themselves. Older adults in the neighbourhood of interest did not directly think of interpersonal factors influencing their eating behaviour, but rather of individual factors or environmental factors. There were, however, several interpersonal factors influencing eating behaviour. Some of these factors seemed partly neighbourhood-specific, and therefore stressed the need to investigate the specific context before developing and implementing an intervention programme. The insights from this study can assist in developing health-promoting strategies for older adults, taking into account the context of the specific neighbourhood.

## Supplementary information

**Additional file 1.** COREQ 32-item checklist for interviews.pdf.

**Additional file 2.** Interview questions.pdf.

## Data Availability

This article includes all data relevant to support the findings by means of (translated) verbatim quotations. Additional information can be requested from the corresponding author [AR]. The raw data as a whole cannot be made publicly available as this type of data can be traced back to individual participants, which would compromise their privacy.
