# Supplementary material for: Interpersonal determinants of eating behaviours in Dutch older adults living independently: a qualitative study
Source: BMC Nutr. 2020 Nov 11;6:55. doi: 10.1186/s40795-020-00383-2 (PMC7656669; doi:10.1186/s40795-020-00383-2)
Supplement: Supplementary file 2 — Additional file 2. Interview questions.pdf. [file 40795_2020_383_MOESM2_ESM.pdf]

Additional file 2 – Interview questions

Topic	Questions to start with:	Ask why! Other follow-up questions:
Introduce yourself	We would like to get to know you a little better. Could you briefly tell us something about yourself?	
Socio-demographics	How old are you?	
	What does your family situation look like?	What is your marital status?<br>Do you have children? And grandchildren?
	Do you live alone or with someone else?	With whom?
Daily life	What is important for you in life? Which things make you happy?	
	Can you tell something about the things/activities you do in a week?	Any hobbies? Physical activity? Member of any association?
Association	When I say 'eating and drinking', what do you think about?	What does eating and drinking mean to you?
Food motives	How do you decide what you eat or drink?	Do you pay attention to:<br>- Price of food?<br>- Quality of food?<br>- What other people eat or drink?<br><br>Are you interested in nutrition?
	Do others pay attention to what you eat? Does that influence your behaviour?	Do you talk with others about eating or drinking?
Traditions & habits	Do you have any habits or traditions regarding eating or drinking? Something that you are used to do?	Are these habits/traditions your own, or do they belong to those of other people around you (for example your family)?
Proper meal	What is a proper meal for you? What makes a meal less proper?	
	Do you think you eat and drink well?	And how is that, when you compare your behaviour to that of other older adults?
Skipping meals	Do you ever skip a meal?	
Eating alone and/or with others & eating outdoors	Do you usually eat alone or together with others?	Do you ever eat with others? If so:<br>- With whom?<br>- When?<br>- Why?<br>- How do you feel about that?
	Where do you usually eat?	Do you ever eat outdoors? Why? Where?
Changes in diet	Has your eating pattern changed in the past years? And since you were 60 years old?	What changed? Why did it change?<br>How did it feel to change the pattern?
Grocery shopping	Who usually does the grocery shopping?	
	Does anyone help you with the grocery shopping?	
	Where do you usually buy your groceries?	How far away is that? How do you get there?
Meal preparation & meal preparation support	Who usually prepares dinner; you or someone else?	Do you ever use ready-to-eat meals? In which situations?
	Do you ever make use of a meal service?	How do you think about that? How did you get there?
Social ties	Are you satisfied with the contact you have with others?	Who are the persons you see or speak to regularly?
Loneliness	Do you ever feel lonely?	When?<br>What do you do in such a situation?<br>What might help you to feel lonely less often?
Final questions	Do you expect any changes in your eating behaviour (in the future)?	Which changes?
	Is there anything left, you would like to share?	
